# Editorial: The Role of Epithelial-Mesenchymal Transition-Related Non-Coding RNAs in Cancer

**DOI:** 10.3389/fmolb.2022.839952

**Published:** 2022-01-21

**Authors:** Rui Cao, Yu Xiao, Xuefeng Liu

**Affiliations:** ^1^ Department of Urology, Beijing Friendship Hospital, Capital Medical University, Beijing, China; ^2^ Department of Biological Repositories, Zhongnan Hospital of Wuhan University, Wuhan, China; ^3^ Human Genetic Resource Preservation Center of Hubei Province, Wuhan, China; ^4^ Wuhan Research Center for Infectious Diseases and Cancer, Chinese Academy of Medical Sciences, Wuhan, China; ^5^ Department of Pathology, Lombardi Comprehensive Cancer Center, Georgetown University Medical School, Washington, DC, United States

**Keywords:** non-coding RNAs, cancer, epithelial to mesenchymal transition, circRNA, lncRNA, miRNA, snoRNA, metastasis

Epithelial mesenchymal transition (EMT) is a cellular biological program that naturally occurs in a wide range of tissue types and developmental stages (Zhang and Weinberg, 2018). During this process, epithelial cells progressively lose their cobblestone epithelial appearance in monolayer cultures to adopt a spindle-shaped, mesenchymal morphology. However, the mesenchymal characteristics are reversible as the resulting more-mesenchymal cells can revert back to an epithelial state in the reverse process, also known as mesenchymal-epithelial transition (MET) (Kalluri and Weinberg, 2009; Thiery et al., 2009). After activation of EMT, the expression of E-cadherin, one of the most important surface epithelial cadherin to the structural integrity of epithelia, is inhibited, resulting in the loss of typical polygonal and cobblestone morphology of epithelial cells. Cells acquire spindle mesenchymal cell morphology and express markers relate to mesenchymal cell status, such as neural cadherin (N-cadherin), vimentin and fibronectin 3 (Dongre and Weinberg, 2019). In recent years, with the progress of research, the progress of EMT can lead to several typical characteristics of malignant progression of cancer cells, including tumor initiation characteristics, motility, transmission ability and resistance to common chemotherapeutic drugs (De Craene and Berx, 2013; Fiori et al., 2019; Mashouri et al., 2019; Pastushenko and Blanpain, 2019). At the same time, many EMT related biomarkers are considered to be specific markers of high malignancy.

In recent years we have seen a rapid development of next-generation sequencing. Some non-coding RNAs (ncRNAs), such as microRNA (miRNA), long non-coding RNA (lncRNA), circular RNA (circRNA) and small nucleolar RNA (snoRNA), which are first thought to be transcriptional noise have now been reported to play important roles in many biological processes including tumorigenesis. However, the role of EMT-related ncRNAs in cancer development remain largely unknown.

Therefore, in this research topic, we are focusing on the underlying crosstalk between EMT processes and ncRNAs in oncogenesis, including competing for endogenous RNA (ceRNA), genetics alteration (RNA interference, mutation), epigenetics regulation (DNA methylation, post-translational modifications of histones) as well as exploring the potential for EMT-related ncRNAs to serve as molecular markers/signatures in the prediction of the tumor patient’s survival, chemotherapy, radiotherapy and immunotherapy response.

Hussen et al. described the influence of ncRNAs including microRNAs and long non-coding RNAs in the EMT process and their application as biomarkers for this process and cancer progression and their potential as therapeutic targets. They mentioned that expression levels of EMT-associated miRNAs and lncRNAs has been linked to the survival of cancer patients. Therefore, it was possible that a panel of EMT-associated miRNAs and lncRNAs predict disease progression and therapeutic response with clinically relevant accuracy. However, there was no consensus set of ncRNAs to facilitate the design of such diagnostic tools as yet, which could be a potential direction worth working for.

This situation had gained considerable progression in the research of some tumors. Takahashi et al. showed us that lncRNA was not only involved in the pathogenesis, invasion and metastasis mechanism of pancreatic cancer, but some specific lncRNA had been used as a diagnostic and prognostic indicator for pancreatic ductal adenocarcinoma (PCAD). At the same time, in the clinical application of tumor therapy, some studies had taken lncRNAs as potential therapeutic tools because they could help promote the therapeutic strategies targeting tumor cells.

In recent years, as immunotherapy has brought a new era to cancer treatment, tumor immune microenvironment has been paid more and more attention by researchers. Liu et al. indicated that the prognostic value of EMT-related lncRNA in colorectal cancer (CRC) patients and explored potential mechanisms and regulatory networks. And their results showed AL591178.1 was a risk factor for the prognosis of CRC patients, which was inversely proportional to the level of immune cell infiltration, P53 pathway, and ECM-receptor pathway.

In addition, other researchers focused on more specific lncRNAs or pathways indicating their role in tumors. Islam Khan and Law demonstrated that silencing of CASC9 contributes to the reduced CRC cell proliferation and migration by regulating autophagy and AKT/mTOR/EMT signaling. And Luo et al. demonstrated that the JAK2/STAT1 signaling pathway was closely involved in the ENZ resistance process induced by CXCR7 derepression, and target inhibition of the CXCR7-JAK2/STAT1-C-Myc signaling pathway might be an important and effective strategy to overcome ENZ resistance in patients with CRPC.

Of course, the role of other kinds of ncRNAs in tumors cannot be ignored. Tanabe combined microRNA to study the role of EMT in tumor progression, metastasis and advanced tumor treatment. And the results by Zhang et al. showed circRNA circ_0001666 acts as an oncogenic circRNA to promote EMT and invasion of pancreatic cancer (PC) cells through sponging miR-1251, and indicated that circ_0001666 could be explored as a potential therapeutic target for PC. This also suggested that circRNA may be a potential tumor therapeutic biomarker.

This Research Topic of articles in the research topic covers the role of various ncRNAs in different tumors. With the deepening of future research, we have reason to believe that the role of ncRNAs in tumor progression, metastasis and invasion will be more and more clear (Anastasiadou et al., 2018; Chan and Tay, 2018; Slack and Chinnaiyan, 2019). At the same time, we also believe that the diagnosis, prognosis prediction and treatment combined with ncRNA will play an important role in pan-cancer treatment in the future (Matsui and Corey, 2017; Nicolas, 2017; Yuan et al., 2020).
